# Re: Preliminary assessment of neck circumference in benign prostatic hyperplasia in patients with metabolic syndrome

**DOI:** 10.1590/S1677-5538.IBJU.2017.0197

**Published:** 2017

**Authors:** Huseyin Besiroglu, Emin Ozbek

**Affiliations:** 1 Department of Urology, Çatalca Ilyas Çokay State Hospital, Istanbul, Turkey; 2 Department of Urology, Istanbul Training and Research Hospital, Istanbul, Turkey

*To the editor,*

We read with great interest the current study written by Akin and colleagues (1) entitled ‘Preliminary assessment of Neck Circumference in Benign Prostatic Hyperplasia in patients with Metabolic Syndrome’. BPH is one of the most common diseases in aging men and despite intense research the exact etiopathogenesis is still under debate. Age and androgen stimuli are the well-known factors for prostate enlargement but multiple partially overlapping and complementary systems (nerve, endocrine, immune, and vascular) as well as local factors are also likely to be involved (2). Scientific interest in the association between anthropometric measurements and prostate hyperplasia has mainly been focused on height, weight and measures of weight adjusted for height, such as body mass index (BMI) with less interest in waist circumference and its ratio to hip circumference. Although published epidemiological data demonstrate that obesity may increase the risks of BPH and LUTS, the quantitative evidence to certify this association is still lacking. Firstly, we congratulate the authors for their great effort in carrying out a study that provides some quantitative results on the potential association of the relatively little used anthropometric measurement, neck circumference, with BPH parameters. Nevertheless, there are some concerns that should be addressed and need further discussion. Contrary to many studies (3-5), the baseline parameters including IPSS, Q max value, and prostate volume were comparable between the two groups (with MtS vs. without MtS) in the current paper. According to the present study, although the baseline BPH parameters did not significantly differ, the patients with MetS had decreased benefit from alpha-blockade drugs. Being prospective, one of the strong attributes of the present study, these results are valuable but the statistical analysis could be misleading as few patients were included, so they should be tested with more data before being used in clinical applications.

We should also address some potential considerations: It is unclear whether waist circumference, BMI, neck circumference or waist-to-hip ratio constitute the best anthropometric parameters for correlating BPH parameters with central obesity. Additionally, these are only measurement tools indirectly suggestive of general body composition or central obesity and their accuracy can easily be affected by ethnical and racial changes. Thus, the outcomes from this study that comprised only Turkish men should not be generalized to those of other races or ethnicities if the effects of the disease processes that lead to the development of LUTS and BPH differ among these groups.

Consequently, this study provides important preliminary results for the potential unfavorable effect of metabolic syndrome in BPH patients treated with alpha blockade drugs. It is obvious that the clinical significance of these results should be tested with further well-designed studies including a larger cohort.
